# Supplementary material for: MetaRibo-Seq measures translation in microbiomes
Source: Nat Commun. 2020 Jun 29;11:3268. doi: 10.1038/s41467-020-17081-z (PMC7324362; doi:10.1038/s41467-020-17081-z)
Supplement: Supplementary file 10 — Supplementary Data 7 [file 41467_2020_17081_MOESM10_ESM.zip › File2/Confidence_High_Taxonomy/422159_out.krona.html]

Javascript must be enabled to view this page.

members
magnitude
magnitudeUnassigned
count
unassigned
taxon
rank

422159\_out

6

2
superkingdom
6

phylum
6
976

200643
6
class

order
6
171549

family
6
815

6
genus
816

457390

SRS020271\_contig\_number\_contig-100\_775.31159
1
species

28116

SRS015960\_contig\_number\_13765SRS098881\_contig\_number\_14332
2
species

817

SRS048981\_contig\_number\_453
1
species

1262750
species
2

SRS055966\_contig\_number\_1044SRS063127\_contig\_number\_1273
